# Case Report: Immune checkpoint inhibitor-induced myositis without elevated creatine kinase

**DOI:** 10.3389/fimmu.2025.1592385

**Published:** 2025-06-25

**Authors:** Klajdi Begaj, Raphael Wilhelm, Alisa Lepper, Maike Kaufhold, Jakob Veeser, Stephan Grabbe, Henner Stege

**Affiliations:** Department of Dermatology, University Medical Center of the Johannes Gutenberg University, Mainz, Germany

**Keywords:** immune-related adverse events (IRAE), myositis, immune checkpoint inhibitors, creatine kinase, melanoma, case report

## Abstract

Immune checkpoint inhibitors (ICIs) have revolutionized the treatment of advanced cancers like malignant melanoma. However, they can lead to a range of immune-related adverse events (irAEs), impacting various organ systems. Among these, myositis is a rare but serious irAE, typically characterized by myalgia, muscle weakness, and elevated creatine kinase (CK) levels. Herein, we report the case of a 58-year-old female with advanced melanoma, who presented a delayed-onset of ICI-induced myositis accompanied by severe muscle weakness. Interestingly, the CK levels remained normal throughout her disease course. Neurological examination, MRI, and electromyography were pivotal in diagnosing myositis. Differential diagnoses, including myasthenia gravis, myocarditis, and paraneoplastic syndromes or idiopathic inflammatory myopathies, were systematically ruled out through clinical evaluation, serological testing, and imaging. The patient responded favorably to high-dose corticosteroid therapy, leading to a gradual improvement of symptoms and no relapse after stopping treatment. This case report emphasizes a multimodal diagnostic approach and underscores the importance of clinical awareness for such atypical irAE presentations.

## 1 Introduction

Immune checkpoint inhibition has emerged as a transformative approach in oncology over the last decade, especially for melanoma patients. CTLA-4 and PD-1/PD-L are two primary targets of immune checkpoint inhibition and play critical roles in downregulating T-cell antitumor responses. The inhibiting of these key regulatory molecules allows the immune system to target and attack cancer cells. Monoclonal antibodies such as ipilimumab (anti-CTLA-4) and nivolumab (anti-PD-1) have significantly improved patient outcomes and survival rates in advanced melanoma (1–3).

While immune checkpoint inhibitors (ICIs) have revolutionized cancer treatment, they can also lead to a spectrum of inflammatory side effects, collectively known as immune-related adverse events (irAEs), which usually affect the gastrointestinal tract (colitis), endocrine glands (e.g., thyroiditis, hypophysitis), lungs (pneumonitis), skin, and liver (hepatitis). These adverse events may lead to discontinuation of ICI therapy and are typically treated with corticosteroids or other immunosuppressive/immunomodulating agents like intravenous immunoglobulins (IVIGs) or infliximab in steroid-resistant cases (4–6).

The underlying mechanisms driving these irAEs are not completely understood, but potentially involve several pathways. These include the direct response of ICI-activated autoreactive T-cells, B-cell-mediated autoantibody production, excessive pro-inflammatory cytokine release and possible alterations in the gut microbiome (2, 7).

Myositis is another rare but serious irAE. Typically, ICI-induced myositis presents with muscle weakness, myalgia, and elevated creatine kinase (CK) levels, reflecting muscle damage (8, 9). Cases without CK elevation are exceptionally rare —yet possible and pose a great diagnostic challenge (9).

In this report, we discuss a rare case of a patient with advanced melanoma who developed ICI-induced myositis without elevated CK levels, highlighting the diagnostic complexities associated with such atypical irAE presentations.

## 2 Case presentation

A 58-year-old caucasian female patient with malignant melanoma (pT1b pN3c cM1d, clinical stage IV) presented with progressive muscle pain and weakness.

**Figure 1:**
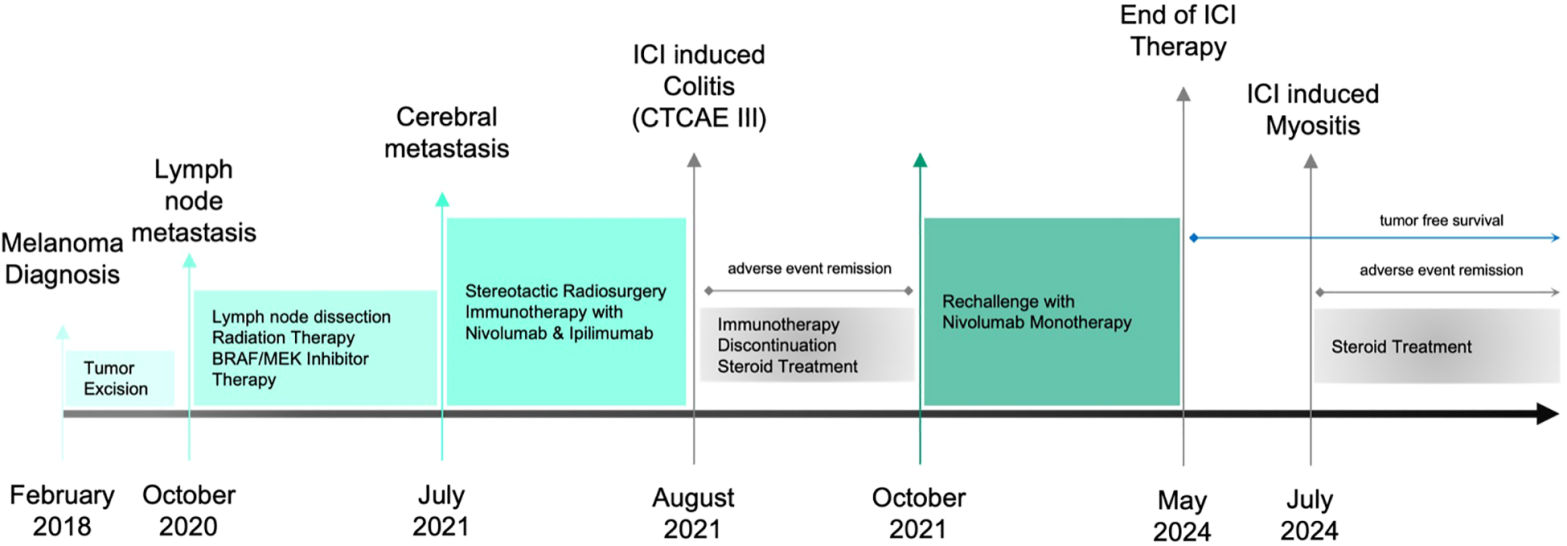
Timeline showing the patient’s clinical course, including diagnoses, therapies and interventions, as well as adverse events and their management.

The primary melanoma lesion had been surgically removed in 2018. Following the detection of axillary lymph node metastasis, the patient underwent lymph node dissection and received targeted therapy with BRAF/MEK inhibitors. Upon developing cerebral metastasis, she was started on combination immunotherapy with ipilimumab and nivolumab. During this dual therapy the patient developed a CTCAE Grade III ICI-induced colitis and after recovery her therapy was switched to nivolumab monotherapy. A timeline of the patient’s history and treatment is provided in **Figure 1**.

Six months before the presentation, the patient began experiencing localized pain in the axillary region, the site of prior lymph node dissection, accompanied by fatigue. At that time, these symptoms were attributed to the ongoing treatment regimen. Following the tumor board recommendations in May 2024, nivolumab therapy was discontinued due to the patient’s tumor-free status, and she was transitioned to routine follow-up.

Approximately two months after discontinuing ICI therapy, the patient developed new-onset myalgia in the hip region, which gradually progressed and extended to the lumbar spine. In addition to pain, she reported muscle weakness in the affected regions, ultimately resulting in gait disturbances. Before the onset of these symptoms, the patient had no history of neuromuscular issues and was fully independent in her daily activities, including walking longer distances and climbing stairs.

**Figure 2:**
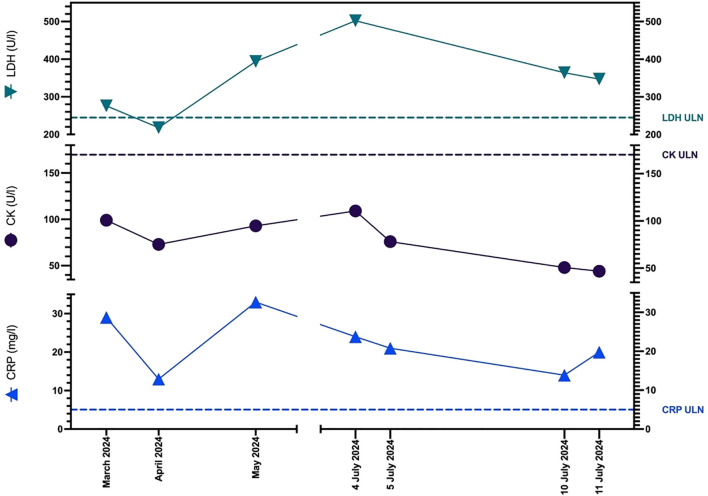
Graph showing patient’s CK, LDH, and CRP levels before the onset of myalgia (March 2024), at the time of presentation and admission (4 July 2024) and on the day of discharge (11 July 2024). ULN, Upper Limit of Normal.

Despite the severity of her symptoms, CK levels remained consistently within the normal range (**Figure 2**). As her condition gradually worsened over the past several weeks, the clinical presentation raised concern for a possible delayed-onset immune-related adverse event, prompting further investigation.

### 2.1 Assessments and investigations

The clinical examination revealed neurological deficits, with significant weakness noted in both the hip and knee flexors. In these compartments muscle strength was reduced bilaterally (graded at 2-3/5), and the patellar reflex was absent on both sides. There were no signs of ocular weakness, such as ptosis or double vision, and no dysphagia was observed. Sensory functions remained intact, and other reflexes were normal, indicating a selective involvement of the proximal lower limb musculature without widespread systemic involvement.

Laboratory tests upon admission (**Table 1**) revealed a normal complete blood count. There was a slight elevation in ALT and AST with normal bilirubin levels. Inflammatory markers showed a mildly elevated CRP and the LDH level was twice the upper limit of normal (ULN). Both CK and Troponin I were within normal limits. The melanoma tumor marker S-100 was slightly elevated.

**Table 1:** Laboratory findings of the patient upon admission. The bold and highlighted values are clinically relevant.

Parameter	Result	Reference Interval
Erythrocytes	5.00/pl	4.0-5.0/pl
Thrombocytes	319/nl	165-270/nl
Leukocytes	6.9/nl	3.7-9.2/nl
Sodium	140 mmol/l	136–145 mmol/l
Potassium	3.8 mmol/l	3.4-4.9 mmol/l
Calcium	2.36 mmol/l	2.15-2.58 mmol/l
CK	109 U/l	30–170 U/l
Troponin I	3.7 pg/ml	<24 pg/ml
LDH	502 U/l	<245 U/l
Total Bilirubin	0.6 mg/dl	0.2-1.2 mg/dl
AST	65 U/l	5–31 U/l
ALT	44 U/l	<35 U/l
CRP	24 mg/l	<5 mg/l
Creatinine	0.57 mg/dl	0.55-1.02 mg/dl
eGFR	102 ml/min/1.73sqm	50–98 ml/min/1.73sqm
S-100	0.15 μg/l	< 0.105 μg/l
ANA	1:160	<1:80
ENA panel	negative	negative
Myositis blot	negative	negative
Systemic Sclerosis blot	negative	negative
Acetylcholine Receptor Antibodies	negative	negative

Autoimmune screening showed weakly positive antinuclear antibodies (ANA) at a titer of 1:160 with a homogenous granular pattern, though subsequent ENA profile tests were negative. Additional autoantibody tests, including a myositis blot, systemic sclerosis blot, and acetylcholine receptor antibodies, showed no abnormalities.

**Figure 3:**
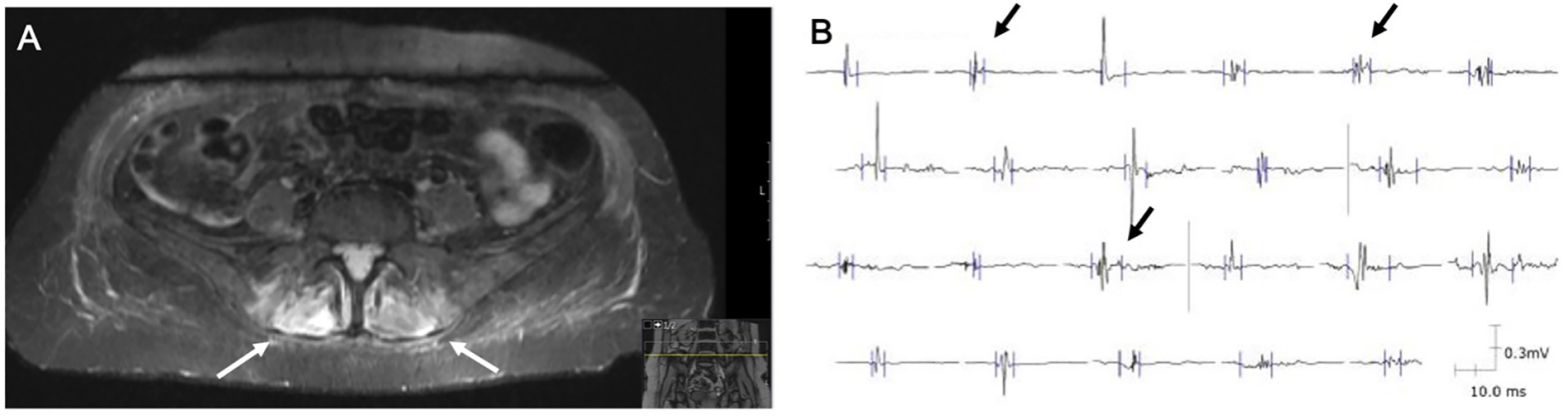
Radiological and electromyography findings leading to the diagnosis of ICI-induced myositis. **(A)** MRI in the TIRM sequence showing hyperintense signals in the autochthonous spinal muscles (white arrows). **(B)** EMG of the right gluteus medius muscle displaying polyphasic, short-duration, low-amplitude motor-unit potentials (black arrows).

MRI of the lumbar spine and pelvis (**Figure 3A**) showed no significant spinal canal stenosis or nerve root compression, but revealed significant hyperintense signals in the turbo inversion recovery magnitude (TIRM) sequences of gluteal and autochthonous spinal muscles. Electromyography (EMG) of the right gluteus medius and vastus lateralis muscles (**Figure 3B**) demonstrated polyphasic, low-amplitude motor-unit potentials of short duration. Both examinations indicated myopathic changes, consistent with the diagnosis of ICI-induced myositis.

### 2.2 Diagnosis, management and follow-up

Considering the previous examinations, we established the diagnosis of delayed-onset of ICI-induced myositis without CK elevation, occurring two months after discontinuing immunotherapy. Subsequently, a high-dose (250 mg) intravenous prednisolone therapy was initiated for three days, followed by a gradual tapering regimen over eight weeks. She was referred for outpatient rehabilitation, which resulted in an improvement of her symptoms. No recurrence of symptoms was observed after tapering and stopping the steroid treatment. At her 3-month and 6-month follow-up visits, the patient remained symptom free with full restoration of muscle strength (graded 5/5) and mobility. She continues under routine surveillance at our skin cancer center, with multidisciplinary input form neurology and oncology teams. Periodic clinical evaluations and laboratory monitoring have shown no signs of relapse.

## 3 Discussion

Our report highlights an unusual presentation of delayed-onset ICI-induced myositis without CK elevation, which, to date, has not been described in detail. After experiencing immune-related colitis, the patient remained nearly two years free of any irAE. During that time the patient followed a treatment regimen with nivolumab monotherapy. Nivolumab is commonly associated with a reduced risk of adverse events compared to combination therapy (3). Apart from the absence of CK elevation, the onset of symptoms after treatment discontinuation made the diagnostic process even more challenging. Despite the unusual presentation, careful consideration of symptoms, laboratory testing, MRI, and EMG ultimately led to the accurate diagnosis and management of this irAE.

When evaluating neuromuscular adverse events linked to immune checkpoint inhibitors, three conditions should be primarily considered: myositis (often involving the cervical and oculo-bulbar muscles), myocarditis, and myasthenia gravis. In rare and severe cases, there may be an overlap syndrome involving all three conditions. In this patient, normal Troponin I levels and a negative cardiological examination excluded myocarditis. Additionally, the negative acetylcholine receptor antibody test ruled out ICI-induced myasthenia gravis (10–13). Other important differential diagnoses to consider in similar clinical presentation include paraneoplastic syndromes and idiopathic inflammatory myopathies (IIMs) such as dermatomyositis and polymyositis (14). The lack of additional cutaneous and systemic features and the negative serology reduced the likelihood of these alternative diagnoses, supporting the conclusion of ICI-induced myositis.

The ANA titer of 1:160, though positive, was deemed clinically insignificant, especially given the subsequent negative ENA blot. Additionally, the absence of myositis-specific autoantibodies is consistent with existing literature, which suggests that ICI-induced myositis tends to be seronegative, lacking the typical autoantibodies found in other autoimmune muscle disorders (15–17).

The pathophysiology of ICI-induced myositis is not yet fully understood, however it is hypothesized that it is primarily a cell-mediated process, with humoral autoimmunity likely playing a minimal role in its pathogenesis. Emerging evidence suggests that enhanced T-cell activation and loss of peripheral tolerance lead to direct cytotoxic effects on muscle fibers. This immune response may involve clonally expanded CD8+ T cells infiltering muscle tissue, accompanied by upregulation of MHC class I expression on muscle fibers. Inflammatory cytokines such as IFN-γ, TNF-α, and IL-6 may further amplify this response and promote tissue injury. This selective targeting indicates an underlying mechanism distinct from common inflammatory myopathies (13, 18, 19).

Our patient’s favorable response to steroid therapy supports the notion that hyperactive T-cell responses were the primary drivers of muscle inflammation, rather than autoantibody-mediated mechanisms. However in cases resistant to corticosteroids, the administration of IVIGs is still recommended as a second-line therapeutic option (14, 20, 21).

Notably, the serum CK levels remained within normal range throughout the disease course. Previous histopathological studies of ICI- induced myositis have shown varying degrees of muscle fiber necrosis, ranging from multifocal clusters to diffuse confluent areas (14, 18, 20). The absence of CK elevation in our patient may be attributable to localized inflammatory responses limited to specific muscle groups of compartments, without necessarily causing widespread muscle lysis and thereby minimizing the systemic enzyme release.

Although CK is a commonly used biomarker for muscle injury, its absence does not definitively rule out myositis. In such circumstances, aldolase, an enzyme also released from damaged muscle cells, could serve as an additional biomarker indicating muscle injury. In addition to aldolase, serum or urine myoglobin levels may also offer further diagnostic utility (13). Including these additional biomarkers into the diagnostic workup may provide more clarity regarding muscle involvement in future cases (22).

However, the diagnostic process for ICI-induced myositis should prioritize clinical findings, imaging, and electromyographic studies, in addition to serum biomarkers. In this case, MRI provided detailed visualization of muscle inflammation, while EMG identified myopathic changes consistent with myopathies such as myositis. Together, these modalities were essential for confirming the diagnosis and guiding the appropriate treatment strategy. Our patient’s condition subsequently stabilized following steroid therapy, yet regular follow-up remains essential. Given the occurrence of two distinct irAEs, one of which with delayed manifestation, she remains at elevated risk for future immune-mediated complications.

### 3.1 Conclusion

This case underscores the importance of remaining vigilant for irAEs, even months after treatment discontinuation. Moreover, our report supports the understanding that ICI-induced myositis may represent a distinct clinical entity, requiring a multimodal diagnostic approach —combining clinical assessment, imaging, electrophysiological studies and laboratory workup— especially when classical biomarkers such as CK are absent. A high index of suspicion, early recognition through cross-disciplinary assessments and tailored management are crucial for optimizing patient outcomes in atypical irAE presentations.

## Data Availability

The original contributions presented in the study are included in the article/supplementary material. Further inquiries can be directed to the corresponding author.
